# Application of Platelet Rich Plasma in Sports Medicine

**DOI:** 10.2478/v10078-011-0076-z

**Published:** 2011-12-25

**Authors:** Krzysztof Ficek, Tomasz Kamiński, Ewa Wach, Jerzy Cholewiński, Paweł Cięszczyk

**Affiliations:** 1 Galen Orthopaedics, Bieruń, Poland; 2 Department of Physiological and Medical Science Academy of Physical Education, Katowice, Poland; 3 Boni Fratres Catoviensis, Katowice, Poland; 4 University of Szczecin, Department of Physical Culture and Health Promotion, Poland

**Keywords:** Platelet-rich plasma, tendinopathy, musculoskeletal injuries

## Abstract

Any new method of treatment is associated with high expectations for its success, particularly if the therapy is based not only on the premise of achieving a symptomatic effect, but also improving functional quality and repairing structurally damaged tissues. Platelet Rich Plasma (PRP) application was shown to be a successful catalyst in the healing process for a wide variety of conditions in animal and human models. However, its use has been controversial due to many types of the PRP definition, optimal concentration, and modalities of implementation. In the qualification of patients for PRP treatment, not only should medical indications be considered, but also the role of participation in therapy with a physiotherapist supervising physical parameters and techniques used during recovery time. Further study is required in order to define optimal handling procedures of PRP injection. Long-term follow up will reveal if the promise of this substance can be realized and implemented to maximize its potential as a therapeutic remedy.

## Introduction

Introducing any new method of treatment is associated with high expectations for its success. This is true particularly when it concerns the field of physical activity, where a return to activity is essential for verifying the method’s effectiveness, especially if therapy is based not only on the premise of achieving a symptomatic effect, but also improving functional quality and repairing structurally damaged tissues. This brings hope to the use of autologous platelets as a source of growth factors (GF). This method, known as platelet rich plasma (PRP) application, is becoming a more widely practiced standard in sports medicine, orthopaedics, traumatology, general surgery, dentistry and cosmetology (Hall et al., 2009; Virchenko and Aspenberg, 2006). The concept of using PRP derived from the patient’s own blood to improve tissue function by rebuilding its morphology and improving its metabolism sets itself apart from conventional means of interaction with tissue and together with tissue engineering, sets a new course for the development of medicine (Creaney et al., 2011).

The natural reaction of tissues to damaging factors is healing. It is a constant process dependent on the passage of time and many other factors like genetics, environmental conditions, lifestyle, hygiene, level of activity, and general health condition. The defensive mechanisms of healing are a reaction to the sudden activation energy or chronic overload of tissues beyond their threshold to adapt and regenerate. Healing is a coordinated series of activation processes: division, chemotaxis, migration and differentiation of many cell types. All phases of healing are directly or indirectly controlled by cytokines and GF. GF affect undifferentiated stem cells (SC), partially differentiated cells such as fibroblasts, or differentiated cells such as fibrocytes. Extremely important in this process is the state of the extracellular matrix (ECM) of the specific organ. This is because it binds to the cell and forms a support for it, in addition to controlling its functions through enzymatic effect by penetrating its biologically active compounds. The most important element of this system appears to be the population of SC stimulated by platelets with GF, because their basic biological task is to regenerate damaged tissues (Casabona et al., 2010; Chen et al., 2010). SC, due to their versatility in transforming and producing various cytokines, seem to be an inexhaustible source of freely-designed biological cells and modulators. As a result, they are the focus of everyone involved in tissue engineering. Improper healing does not restore the ability of tissue to function, accelerating the aging of tissues and may also play a role in biochemical disturbances in lipid peroxidation, resulting in damage to DNA, amino acids and proteins (Anitua et al., 2004).

Sudden activation energy in the form of an external injury biologically damages healthy tissue. In case of overload syndrome, what must be dealt with is the weakening of tissue quality due to chronic excessive burden of cumulative effects and fatigue. This can be attributed to the concentration of waste products and the lack of their removal and it can alter the structure of collagen fibers and their fragmentation, as well as cause spatial disorientation. Dehydration and changes in the vasculature and the supply of oxygen can cause an inflammation, whereby local cellular infiltration decreases mechanical parameters of the tissue, and as a result, leads to interruption of their continuity (Langberg et al., 1999).

The inception of PRP treatment is an attempt to mimic the natural response of living organisms to tissue damage. In the induction phase, the energy of the destructive effect on tissue homeostasis causes blood vessel rupture and extravasation of blood. On this basis, vasoactive mediators involved in the stimulation of platelets for clot formation are activated. Platelet molecules release platelet GF, which initiate a chain reaction of proliferation, cell differentiation and synthesis of the ECM. The use of PRP is not only to repair damaged structures, but also to create a biological model which encourages tissue regeneration by improving the metabolic functions of those structures. The success of this method of treatment depends not only on the quality of the local surface tissue, but also the global tissue and movement quality. A result of the natural aging process of tissue is also a weakening of its regeneration potential, which may be reduced through persistent or dynamic stimulation of the negative impulse, exceeding the tissue’s strength. This inspired the search and introduction of a positive catalyst for a treatment, which seems to be PRP (Dohan Ehrenfest et al., 2009).

## Platelets

Platelets are the smallest fragments of the blood cells derived from fragmentation of precursor megakaryocytes. Megakaryocytes are derived from the SC, myelopoiesis line CFUGEMM, which is derived from a pluripotential hemopoietic SC CFU-M (Antiua et al., 2004). Platelets are 3,6 ± 0,7μm across and 7,06 ± 4,85μm^3^ volume. Their number in the blood of a healthy person varies from 150 thousand up to 400 thousand per 1mm^3^. Platelets do not have a nucleus or DNA. During platelet activation, granule exocytosis occurs, which involves a molecular mechanism identical in other secretory cells of the body. The platelets contain more than sixty different biologically active substances that are involved in processes of tissue regeneration, angiogenesis, epithelialization, proliferation and differentiation of osteoblasts, and in the synthesis of collagen. A detailed description of these platelet factors and their functions are shown in table nr 1 (Kon et al., 2010).

## Methods of obtaining platelet rich plasma

Platelet plasma containing GF is obtained through centrifugation of whole or peripheral blood, using samples and a centrifuge. Sets of sterile containers from different manufacturers are prepared beforehand. They allow platelet plasma to be easily and quickly obtained, even in an outpatient department. The process of acquisition is based on centrifugation at different speeds (according to manufacturer recommendations) collected from anticoagulant whole blood, and occurs as a result of separating red cells, PRP, and platelet poor plasma, PPP. The separation of individual components from one other occurs automatically using special devices, or is done manually (Marx, 2001; Mazzucco et al., 2009).

Different times and centrifugation speeds recommended by the manufacturers of such kits affect the composition of platelet concentrations containing GF. The basic parameter which should be carefully noted is the percentage of platelet recovery, meaning the number of platelets remaining after separating the remainder of centrifuged blood, compared to the number contained in the volume taken from the patient’s whole blood. It demonstrates the effectiveness of the system in the separation of platelets. Another important parameter is the platelet concentration, given that the quantity of plasma from which the platelets was acquired is suspended. This is important because it determines the volume of product obtained from a given method and allows the best method for relevant clinical needs to be selected (Virchenko and Aspenberg, 2006; Chomicki-Bindas et al., 2010).

The definition of PRP is still a subject of debate. As stated by Marx in 2001, the number is 1 million platelets per microliter, which is a concentration 4–5 times higher than that of whole blood. According to the author, this is a working definition, resulting from the proven clinical effects at these concentrations (Marx, 2001). However, Anitua writes about a number above 300 thousand platelets per microliter, also citing favorable results in clinical applications of such formulations obtained previously. The product obtained with their method is called Plasma Rich in GF (Antiua et al., 2004). Other authors used preparations with a number of platelets up to 14 times in excess of the numbers in whole blood (Franchini et al., 2005).

Currently, commercial systems generate platelet concentrations containing a number of platelets per microliter 4 to 11 times higher than in whole blood, (the volume obtained usually does not exceed a few milliliters) (Hall et al., 2009). It is believed that the effectiveness of blood platelets that contain GF depends on the concentration of platelets and GF, however, there is a lack of data defining the optimal concentration to achieve the desired effect in vivo in humans, and therefore, there are no clear reasons to choose one particular method (Kawasumi et al., 2008). Moreover, the role of plasma proteins in obtaining the final clinical result is not fully understood and perhaps they are responsible for the beneficial clinical effects even at lower platelet concentrations (Chomicki-Bindas et al., 2010).

## Clinical applications of PRP

Platelets are an important reservoir of GF in the human body and carry out important functions in the coagulation process, immunology response, and tissue healing. Tissue healing is a complex process in which complicated interactions take place between connective tissue cells, epithelial cells, cells of the immune system, and blood morphotic bodies. The beginning of healing is often the clot that forms at the site of an injury in both soft tissue and bone. Thus, for more than two decades, interest has grown in the blood as a source of substances that could potentially speed up the healing process (Creaney et al., 2011; Kon et al., 2010). Along with platelets’ activity as GF, they also act as messenger substances, elements in the majority of processes occurring in the tissues, such as those responsible for proliferation, differentiation, chemotaxis and tissue morphogenesis (Vos et al., 2010). They participate in autocrine, paracrine, and endocrine signaling. After being released from the places where they are collected, platelet alpha – granules TGF-α, for example, bind to surface receptors on target cells by running relay systems, transcribing mRNA, secreting corresponding proteins, etc. All of these processes are regulated reflexively by adhesive molecules and other GF (Lacci and Dardik, 2010).

PRP containing GF is applied in clinical practice in various ways. The most common is the formation of a clot from platelets after the addition of thrombin and calcium ions. It has advantages in its simple local application and persistence at the injection site. Another method uses a combination of platelet plasma and allogenic bone transfer or marrow blood cells. These methods require a sterile operating room. In practice, ambulatory blood platelet plasma is administered in liquid form, using a local injection (Dohan Ehrenfest et al., 2009; Lacci and Dardik, 2010).

## Application of platelet rich plasma in orthopaedics and sports traumatology

Within the locomotor system, PRP is used for the treatment of soft tissue and bone. The influence of various GF on the healing process of connective tissue components is quite well known. PRP containing GF is also used in sports medicine, as sport very often leads to injuries of tendons, ligaments and muscles. In this area of medicine especially, prompt and effective treatment is expected. As demonstrated in Hall’s overview of the therapeutic methods in Europe and North America, the beneficial effects of PRP have been confirmed (Hall et al., 2009). Tennis elbow, golf elbow, Achilles tendon damage and plantar fasciitis, rotator cuff syndrome, adductor muscle enthesopathy, jumper’s knee, and runner’s knee, are just some of the diseases entities in which PRP has been successfully employed (Wright-Carpenter et al., 2004; Alfredson, 2005; Hammond et al., 2009; Peerbooms, 2010).

Due to the anatomical and functional differences between tendons, ligaments, joint capsules and muscles and their attachment to bones, the PRP injection site varies. Also variable is the response of tissues to mechanical stimuli acting on them in the form of external force. External loads affect the structure of collagen and the ECM. Activation of external loading changes the conditions of cellular tension and induces cellular interaction. Dynamic transduction in particular, affects the regulation of cellular protein synthesis. A response to the action of external forces is also ECM regeneration. Administration of PRP affects the active cellular tension, ECM and collagen structure (Dohan Ehrenfest et al., 2009).

In the treatment of high-performance athletes, the use of PRP is very popular in Achilles tendon pathology, while maintaining its continuity or its partial termination (Filardo describes the beneficial effect of PRP, even with partial damage to the Achilles tendon, when used as a treatment in combination with unloading, immobilization and rehabilitation procedures) (Filardo et al., 2010). Achilles tendon injuries occur quite often in professional sports. 30% to 50% of all injuries are sports-related tendon injuries (Sharma and Maffulli, 2005). Occupational physical activity in sport, dance, or the military increases the likelihood of Achilles tendon pathology, resulting not only from the load endured, but also due to a greater likelihood of structural and functional degradation in other neighboring and distant parts of the musculoskeletal system. Disorders of the neuromuscular control mechanism may be due to improper work habits and fatigue (Kannus and Natri, 1997). Achilles tendon rupture is usually 4 – 6cm above the final attachment mostly in black males between the ages 30 – 45 years (Davis et al., 1999; Owens et al., 2007). tendon is related to the mechanism of acceleration and deceleration. The most of the serious Achilles tendon injuries are associated with sport (Sharma and Maffulli, 2005). Those most predisposed to Achilles tendon damage take part in running, badminton, squash or cold weather training (Milgrom et al., 2003; Fahlstrom et al., 2002). Apart from the specific sport discipline, the surface and the type of footwear are also important (Kujala et al., 2005).

More than 90% of the damage to the Achilles Tendon stretching resistance is directly proportional to its thickness and collagen fiber content. If the physiological cross-section of the tendon is 1 square centimeter, it has a stretching resistance varying from 500 to 1000 kg (Sharma and Maffulli, 2005). The Achilles tendon receives a load stress 3.9 times body mass during walking and 7.7 times body mass when running. While jogging, the Achilles tendon takes on a force of 9kN which corresponds to approximately 12.5 times body mass. Whether Achilles tendon rupture is partial or total is determined in part by the force, but mainly by the speed of the injuring act (Giddings et al., 2000).

Two thirds of Achilles tendon injuries in athletes are accompanied by foot overpronation (Vos et al., 2010). Excessive volume and intensity during training are also important causes of tendinopathy. Repeated excessive overloads lead to degeneration of its fibers. Tissue response is dependent on the size of the load. If the damage cannot be repaired, then the strength of the tendon is reduced and it may rupture (Doral et al., 2010). Degeneration results in micro tears, which leads to cellular hypertrophy. In addition, concomitant hypoxia, malnutrition, hormonal changes, chronic infection and pain contribute to the deepening pathology. Achilles tendon tendinopathy is often accompanied by pain (Mohr et al., 2005).

The key to the emergence of degenerative changes in the tendon fibers is a disruption of homeostasis, vascular perfusion. Avascularity, which negatively affects the metabolism of tendon fibers, reduces the values of elasticity, contractility, preload and regeneration by increasing the accumulation of waste products. These observations were confirmed at the level of the microstructure by Kjaer’s microdialysis. In states of overload of the Achilles tendon, both bruising and mukoid degeneration might be observed (Kjaer et al., 2000). This leads to the creation of mid-portion fibrosis or cysts filled with fat or mucoid that do not affect the continuity of collagen fibers. This is the kind of degeneration of the tendon tissue which an imbalance between synthesis and breakdown of the tendon ECM (Langberg et al., 1999).

The structural specifications and functions of the Achilles tendon require the accurate identification of the PRP injection zone. Other than manual examinations, this is done mostly using ultrasound, which by means of dynamic imaging, precise pathology detection, and ability to measure blood flow through the vessel, makes it easier to control and distribute the application of PRP. For these reasons, ultrasound diagnosis is more highly regarded than magnetic resonance imaging of Achilles (Kahn et al., 2003). An increase in activity and the number of blood vessels visible on the surface in the ultrasound imagery is a compensatory response to blood flow deficit within the tendon (Peers et al., 2003; Alfredson et al., 2003). Neovascularization is also blamed for increased tissue tightness in the already swollen degeneration zone (Sterkenburg et al., 2010). Additional vascular constriction increases the mechanical conflict with peripheral nerve endings, innervating the Achilles tendon and the surrounding tissue. Clinical observations were also confirmed by biopsy tests of this area. The results of these studies also confirmed the impact of structural changes to fibers and vessels on neurotransmission (Ackermann et al., 2002). In order to reduce neurotransmission, the developers of the method introduced measures to reduce the overgrown network of blood vessels (Pufe et al., 2005).

To choose the timing and injection zone of PRP it is helpful to classify the degree of change in the structure of the tendon. These classifications emphasize the distinction between peritenon, or synovial inflammation, and increasing involvement of the tendon substance as a likely reflection of the failure to adapt to physical load, and emphasize the variable stress responses in the tendon structure (Sharma and Maffulli, 2005; Vos et al., 2010). These categories are:

1. Peritenonitis (paratenonitis, tenosynovitis): inflammation of the peritenon
2. Peritenonitis with tendinosis : tendon sheath inflammation associated with intratendinous degeneration
3. Tendinosis: degeneration in the tendon itself to due to cellular hypotrophy
4. Tendinitis: asymptomatic degeneration of the tendon with disruption and inflammatory repair response. A commonly proposed name for the tendon pain problems is tendinopathy.

These listed categories of structural change differentiate the potential sources of discomfort and facilitate their treatment. Locating the problem makes it easier to administer treatment at an earlier stage, before PRP is applied. The use of PRP should be considered as a secondary step of treatment, occurring after or in association with physiotherapy, eccentric training and manual therapy, but prior to surgery, or in order to avoid such procedures as open tendon cleaning or the more widely used Achilles tendon tendoscopy.

PRP usage is increasing in the treatment of commonly occurring enthesopathy, a discomfort in the area of tendons, ligaments and articular capsule attachements, in both professional sports, as well as in recreational physical activity. So far, medical nomenclature has described these conditions as (–itis): epicondylitis, fascitis, and capsulitis, all of which denote the decidedly inflammatory nature of the disease (Sharma and Maffulli, 2005; Magra and Maffulli, 2006). Studies prove that a biopsy of tissue inflammation within the tissues is very brief and only in the initial phase. The subjective experience of these symptoms is determined by the type of tissue degeneration in avascularity (Pufe et al., 2005).

Such a definition of tissue morphology increases the likelihood of effectiveness of treatment through the use of PRP in the damaged area. The use of PRP is statistically proven to be an effective alternative to topical glicocortysteroids —whose effectiveness is only symptomatic (Peerbooms, 2010). Moreover the main activity of steroids is anti-inflammatory, so in stages of degeneration without the inflammatory changes, its use is unjustified. Nonsteroidal Anti-Inflammatory Drugs (NSAIDs) administered symptomatically have a similarly limited efficacy when used at a time when there are no longer any local inflammatory changes. Even though tendon biopsies having shown an absence of inflammatory cell infiltration, anti-inflammatory agents are commonly used (Magra and Maffulli, 2006).

The increasing use of PRP has also surfaced in the practice of knee surgery. The purpose of PRP application is usually to increase the efficiency of healing grafts in anterior cruciate ligament reconstructive knee surgery. Authors using PRP emphasize the benefits of PRP in reconstructive surgery, compared with those in which concentrated platelets are not used (Silva and Sampaio, 2009).

The success of the reconstruction shows the healing graft in the tunnels of the femur and tibia, as well as revascularization and a reconstruction graft in the same direction as the bone structure inside the bone channels and ligamentation inside the joint. These processes are accelerated by the use of PRP, which results in more intensive rehabilitation and a lower risk of inflammatory complications, as well as a faster return to mobility and shortened professional absence (Kawasumi et al., 2008; Hall et al., 2009). A new use is the application of PRP in order to revitalize the graft employed in anterior cruciate ligament reconstruction in the case of its failure. If the continuity of the graft structure is maintained, albeit with weakened mechanical integrity during the arthroscopic procedure, PRP is applied to the zone within the joint which is most vulnerable to subsequent damage (Silva and Sampaio, 2009). In arthroscopic surgery, attempts to use PRP in the treatment of articular surface damage have also been made. Sanchez described the arthroscopic replantation of the articular surface portion of a medial femoral condyle exceeding 2cm in diameter, noting the acceleration of healing of articular cartilage (Sanchez et al., 2003). Whereas, Kon et al. showed that after administration of PRP inside the joint, patients with pain and swelling of the knee experienced significantly decreased pain, improved knee function and greater quality of life (Kon et al., 2010). Clinical trials are being made to prove the effectiveness of PRP in the treatment of degenerative arthritis, and as a healing agent in the implants used in knee and hip joint replacement surgery (Kon et al., 2010). The influence on bone regeneration of platelet concentrate application (often enriched with mesenchyme marrow cells) is visible in the improved quality and speed of bone healing, as well as in complicated fractures where an infection has appeared. Franchini et al. used platelet concentrate in fractures, pseudoarthritis, skeletal reconstructions, in joint replacements, as well as fibrous dysplasia and bone inflammation (Franchini et al., 2005). Kawasumi et al. describe the use of PRP in the treatment of chronic inflammations of the bones (Kawasumi et al., 2008). In most cases, they observed permanent healing of wounds and no recurrence of infection for at least a year. Bielecki et al. confirmed the bacteriostatic and bactericidal properties of PRP in the treatment of pseudoarthrosis and delayed bone adhesions (Bielecki et al., 2007).

### Platelet rich plasma application in other fields of medicine

PRP has long been known as an effective method of treatment in many areas of medicine. It occupies a special place in the treatment of difficult to heal chronic skin ulcers. These ulcers may result from trauma, burns, venous and arterial circulatory disorders, microcirculatory disorders in diabetic and uremic patients, all characterized by a long process of difficult treatment. Consequently, reports of successful treatment with PRP seem very promising (Chen et al., 2010).

Almdahl et al. described the beneficial effects of PRP in the treatment of ulcers caused by the collection of the saphenous vein during coronary artery bypass surgery (Almdahl et al., 2011). PRP has also been used in ophthalmology. Good results were obtained in the treatment of permanent corneal epithelium damage in patients with dry eye syndrome (Lopez-Plandolit et al., 2010).

Intensive examination of PRP has facilitated understanding of the composition and activity of the growth factors present in platelet granules. Due to their regenerative and biostimulating properties, PRP has been used in a trial for the treatment of neurological disorders. In turn, Shen extends the hypothesis that, since receptors for cytokines released by platelets are on the surface of the peripheral nerves, they are also present in the central nervous system and therefore, PRP use could be attempted in the treatment of diseases of the central nervous system, such as: Alzheimer’s, Parkinson’s disease, stroke, and amyotrophic lateral sclerosis (Shen et al., 2009).

A new direction of PRP use is in tissue engineering. New tissue produced from the bioresorbable materials often associated with the saturation of cells with GF or SC, which adhere to scaffold bases, inducing biological functions in the shape of the missing bone or cartilage. In the body, blood clots naturally fulfill the role of scaffold upon which GF operate (Ip and Gogolewski, 2007; Casabona et al., 2010).

## Summary

The success of applying platelet concentrate (PRP) in many fields of medicine is proven. Therefore, this effective form of therapy still requires improvement by clarifying the parameters of an optimal concentration and specifying the half-life decay of active substances, how they work, and their relationships. In increasing the concentration of centrifuged platelets, the linear growth of their activity has no significant effect (Creaney et al., 2011; Kon et al., 2010; Vos et al., 2010). One active ingredient may cause many different effects. Therefore, the multidimensional nature of platelet GF activities means probable effects can only be predicted and not with any certainty. Further analysis is needed to understand the cellular responses in the injection area, which regulate and control the expression of PRP-driven processes affecting the results of treatment (Dohan Ehrenfest et al., 2009).

In the lamellar granules there are more than 60 different biologically active substances, which have potential utility in PRP treatment. Beyond the well-known hemostatic function, platelets release substances that help with tissue repair, accelerate angiogenesis, and alter the inflammatory response. The relatively easy means of obtaining rich plasma and its anti-allergy and anti-bacterial nature are great advantages, as is emphasized by many authors (Bielecki at al., 2007; Mazzucco et al., 2009).

Administering treatment transdermally allows healing of damaged tissue on the post-traumatic hematoma scaffold, without external interference in humoral mechanisms, and without violating the system’s natural defense response to the destruction. The disadvantage of this method is a lack of direct, objective control over the healing quality. Other than subjective sensation, there remains the reference of indirect diagnostic imaging. Ultrasound imaging techniques and MRI are certainly less reliable than direct tissue biopsy, which is, however, impossible to perform routinely, but rather only in exceptional circumstances of clinical research with the approval of an ethics committee (Kjaer et al., 2000; Mohr et al., 2005).

Synergistic increases in the effectiveness of PRP are possible not only through the use of preparatory forms such as manual therapy, shock wave therapy, physical therapy for the essential treatment, but primarily through the identification of local conditions - the quality of tissues and their use, blood supply and innervation, the structure of collagen, previous damage and the effects of previous treatment, and global conditions - state of mobility of neighboring components with mutual influence on each other, bodily posture, muscle chains, fascia trains, extremities axis, mobility and stability of joints (Doral et al., 2010).

In treatment requiring the patient’s participation, it is principal to establish the basis for cooperation with a mutual commitment to positive results. In the treatment strategy for patients with damage within the locomotor system, it is crucial to take advantage of the time of reduced physical activity to improve the patient’s understanding of the risks involved with inappropriate exercise, in order to maintain targeted effort to achieve physical fitness and improve preventative measures to avoid injury to other sections and elements of locomotion. A lack of self-assessment, an overestimation of one’s own capabilities, and neglect of elementary rules of movement often leads to permanent tissue dysfunction. In the qualification of patients for PRP treatment, not only should medical indications be considered, but also the role of participation in therapy with a physiotherapist supervising physical parameters and techniques used during recovery time.

The popularization of physical activity is often dictated by the aggressive commercialization of products necessary to engage in sport and individuals must not be deprived of the basic forms of education and injury prevention. The synergy of these two factors can reduce the risk of injury and enhance the quality of motion.

Despite the lowering prices of PRP sets, they are still expensive. An indirect attempt to solve this problem consists in obtaining the PRP through centrifugation without the use of proprietary separators which yield a standardized concentration and PRP volume. A negative aspect of this approach is the lack of accurate knowledge of the obtained platelet concentrate’s parameters in the absence of a PRP set, as well as the lack of both reproducibility and uniformity of procedural methods (Virchenko and Aspenberg, 2006).

To achieve a compromise between the financial costs of PRP and the desire to utilize the benefit of platelets, full blood can be used. The down side of this approach is the smaller concentration of both platelets and active ingredients than that of a centrifuged preparation. However, economic profit can go hand in hand with skilful and therapeutic use, which is evidenced by the randomizing analysis (Edwards and Calandruccio, 2003).

Activities related to the application of full blood or PRP preparations are strictly sanctioned not only by the aseptic conditions, but also in case of athletes licensed by the World Anti-Doping Agency (WADA). Allowing the use of blood products for the treatment of tissue and topical application has been discussed over the years, and there is a lack of clear guidelines for the next step. Regulations published by WADA formalize the use of these modern methods of treatment, specifying the intramuscular application be maintained according to the required procedures of Therapeutic Use Exemptions (TUE) (WADA, 2010; Engebretsen et al., 2010).

## Figures and Tables

**Pic. 1:**
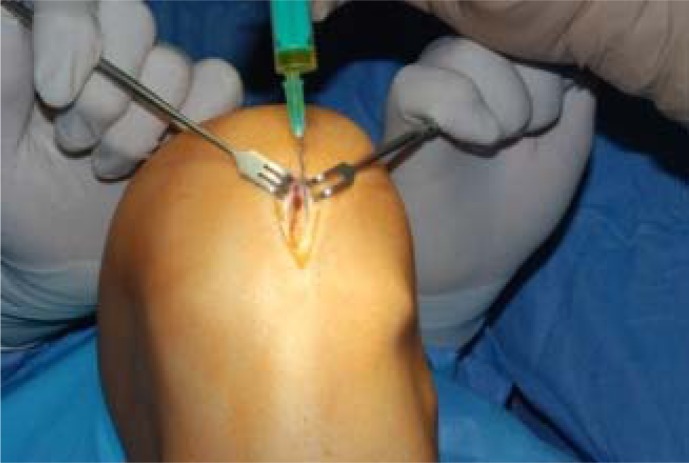
“Jumpers knee”. PRP application in open surgery

**Pic. 2:**
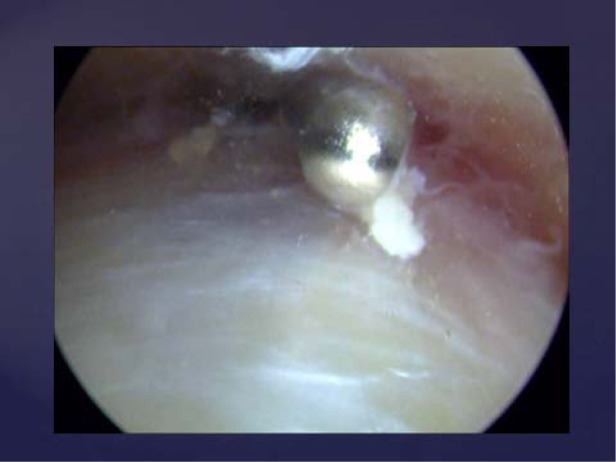
Achilles tendoscopy. Shaving of the soft tissue adhesions

**Pic. 3:**
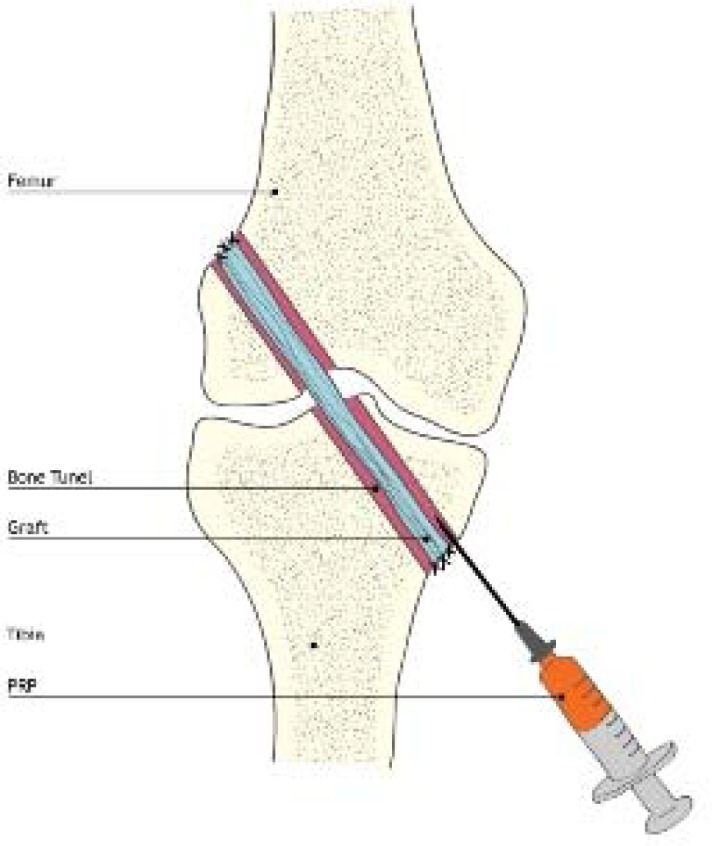
PRP application within the grafted tendons and bone tunnels

**Table 1:** Platelet growth factors

Factor	Parameter
EGF epidermis growth factor	Stimulates the proliferation of epithelial cells, fibroblasts, is a chemoattractant for these cells, stimulates epithelialization, and affects the synthesis and metabolism of the extracellular matrix.
PDGF platelet-dervied growth factor	Both isoform A and B are powerful myogenins for fibroblasts, smooth muscle cells of arteries, endothelium and epithelium. TGFβ activates, stimulating neutrophils and macrophages, mitogenesis, collagen synthesis, and angiogenesis.
TGF-α transformative growth factor alpha	Similar to EGF, binds to the same receptor, stimulates the growth of mesenchymal cells, endothelial and epithelial cells, but also factors in its development. Affects bone formation and regeneration.
TGF-β1 transformative growth factor beta	Stimulates chemotaxis of fibroblasts, collagen synthesis, reduces scarring of the skin, inhibits the growth of epithelial cells, fibroblasts, nerve cells, keratinocytes, and hematopoiesis. Acts in opposition to EGF, PDGF, aFGF, bFGF. It inhibits DNA synthesis in human fibroblasts and control of apoptosis.
KGF keratinocyte growth factor	Strongest for keratinocyte growth factor, is involved in wound healing through proliferation, differentiation, angiogenesis and cell migration, additionally is a myogen/myocyte for many epithelial cells with the exception of endothelial cells and fibroblasts. Increases collagen production by creating a support substance for the skin.
a-FGF or FGF-1 fibroblast growth factor, acidic	Participates in the processes as those above, is a myogen for skin keratinocytes, fibroblasts and endothelial cells of skin.
b-FGF or FGF-2 fibroblast growth factor, basic	Stimulates KGF production, angiogenesis, wound contraction, collagen and matrix synthesis.
VEGF/VEP vascular endothelial growth factor	Stimulates endothelium in large blood vessels, a protein with strong features of angiogenesis, initiates the regeneration of blood supply, causes degradation of metaloprotein synthesis in interstitial collagen types 1, 2 and 3
CTGF connective tissue growth factor	Initiates proliferation, migration, and curling up of vascular endothelial cells, proliferation and differentiation of osteoblasts, mineralization of the matrix.
GM-CSF lub CSF α granulocyte and macrophage colony	Proliferates and differentiates osteoblasts, the synergism of erythropoietin in the proliferation of bone marrow stem cells, is a strong chemoatractant for neutrophils, increases expression of selectin, integrins and adhesion molecules of the immunoglobulin superfamily. Growth factor for fibroblasts, it accelerates angiogenesis, inhibits keratinocyte proliferation and growth, participates in the post burn reactions.
TNF-α tumor necrosis factor	Growth factor for fibroblasts, it accelerates angiogenesis, inhibits keratinocyte proliferation and growth, participates in the post burn reactions.
IGF insulin-like growth factor	Growth factor for normal fibroblasts, enhances synthesis of collagenase and PGE 2 in fibroblasts, regulates the metabolism of articular cartilage through an increased synthesis of collagen and matrix osteon.
IL-1β interleukin 1β	Inhibits the growth of endothelial cells and hepatocytes, activates osteoclasts (albeit at low concentrations, promotes new bone growth) enhances infection and collagenase activity. Enhances the infection processes in the skin and also strongly stimulates fibroblasts.
IL-6 interleukin 6	Mature fibroblasts and macrophages produce IL-6, which stimulates the growth of fibroblasts and collagen production, pro infectious cytokine. In the form associated with blocking protein, inhibits the growth of fibroblasts and collagen production, IL-8 supports angiogenesis and is mitogenic for epithelial cells.
IL-8 interleukin 8	
